# Cubic Nonlinearity of Graphene-Oxide Monolayer

**DOI:** 10.3390/ma16206664

**Published:** 2023-10-12

**Authors:** Tikaram Neupane, Uma Poudyal, Bagher Tabibi, Wan-Joong Kim, Felix Jaetae Seo

**Affiliations:** 1 Department of Chemistry and Physics, The University of North Carolina at Pembroke, Pembroke, NC 28372, USA; tikaram.neupane@uncp.edu (T.N.); uma.poudyal@uncp.edu (U.P.); 2 Advanced Center for Laser Science and Spectroscopy, Department of Physics, Hampton University, Hampton, VA 23668, USA; bagher.tabibi@gmail.com; 3 K1 Solution R&D Center, Geumcheon-gu, Seoul 08591, Republic of Korea; kokwj@daum.net

**Keywords:** one-photon transition, two-photon transition, 2D materials, nonlinear absorption, nonlinear refraction

## Abstract

The cubic nonlinearity of a graphene-oxide monolayer was characterized through open and closed z−scan experiments, using a nano-second laser operating at a 10 Hz repetition rate and featuring a Gaussian spatial beam profile. The open z−scan revealed a reverse saturable absorption, indicating a positive nonlinear absorption coefficient, while the closed z−scan displayed valley-peak traces, indicative of positive nonlinear refraction. This observation suggests that, under the given excitation wavelength, a two-photon or two-step excitation process occurs due to the increased absorption in both the lower visible and upper UV wavelength regions. This finding implies that graphene oxide exhibits a higher excited-state absorption cross-section compared to its ground state. The resulting nonlinear absorption and nonlinear refraction coefficients were estimated to be approximately ~2.62 × 10^−8^ m/W and 3.9 × 10^−15^ m^2^/W, respectively. Additionally, this study sheds light on the interplay between nonlinear absorption and nonlinear refraction traces, providing valuable insights into the material’s optical properties.

## 1. Introduction

The successful extraction of a graphene single layer through mechanical cleavage in 2004 has ignited a wave of research interest in the fascinating world of two-dimensional (2D) materials [1]. Graphene’s exceptional Kerr effect and high nonlinear absorption coefficient make it a highly promising material for various applications in the field of optoelectronics [2,3]. However, the high nonlinear absorption coefficient was accompanied by a zero bandgap through the strong two-photon absorption (TPA) process, which might not only be from the two-step excitation but also due to an undesired free-carrier absorption (FCA) and free-carrier dispersion (FCD) [4]. On the other hand, graphene oxide (GO) has been recognized as one of the growing two-dimensional materials due to its broadband optical effect through the tunable bandgap [5,6]. The content and location of oxygen-containing groups obviously influence the optical and electronic properties [7,8]. These reduction processes transform GO from an insulator to a semiconductor and to a metal-like state, in the form of graphene. In addition, GO is a hydrophilic and water-soluble material due to the existence of the oxygen-containing group which makes the fabrication easier.

The third-order (cubic) nonlinear optical properties of GO are stable under high-power illumination [9], which makes GO a strong candidate for the optoelectronic applications such as pulse compression, mode-locking to Q-switching, optical limiting (OL), and all-optical switching [5,10,11,12,13,14,15,16,17,18,19,20,21,22,23]. The study of third-order optical nonlinearity, which includes the coefficient of nonlinear absorption (NLA), nonlinear refraction (NLR), and their polarity as well, reveals its prospective technical applications in optoelectronics. For instance, materials exhibiting negative nonlinear absorption (NLA) coefficients, characterized by saturable absorption (SA), find application as crucial Q-switching elements in lasers [24]. Conversely, materials featuring positive NLA coefficients, demonstrating reverse saturable absorption (RSA), are well suited for applications such as two-photon microscopy and optical limiters [25]. To study such an enthralling phenomenon, the “z−scan technique” has been used to investigate both the polarity and magnitude of NLA and NLR coefficient [16,26]. Kang et al., revealed that GO exhibits strong and broadband nonlinear optical (NLO) properties for the optical power limiting in a wide spectral range of wavelengths [27]. It obviously meets the demands for emerging photonic applications to overcome the OL behavior at certain wavelengths via metallic nanomaterials (zinc ferrite nanoparticles [28], gold clusters [29]). In addition, the NLO response in a wide spectral range exhibits SA at the short wavelength and RSA at the longer wavelength, which may result in a varying magnitude of third-order susceptibility based on the wavelength-dependent nonlinear optical transition [27]. The magnitude and polarity of third-order nonlinearity are modified using excitation processes such as resonant and non-resonant nonlinear processes. As an example, resonant excitation yields a relatively greater magnitude of nonlinearity compared to non-resonant excitation. However, it is worth noting that resonant excitation demands a longer duration within the nonlinear process compared to its non-resonant counterpart [30]. The literature review divulges that the few layers of GO dispersion and reduced GO (rGO) are good candidates for the optical power limiting at the 532 nm wavelength [31,32,33,34]. Also, GO nanosheets dispersed in DI water demonstrated the broadband NLO and its prospective application in optical power limiting at the 1064 nm wavelength [35]. The tunable OL properties of GO in ethanol solution were studied at 1550 nm as well [36]. In moving towards the application of GO as an optical power limiter, the characterization of third-order optical susceptibility (*χ*^3^) is a pivotal parameter that is directly related to the coefficients of NLA and NLR. Additionally, the imaginary *χ*^3^ articulates the information about the SA and RSA to identify the possible application as an optical power limiter.

Ebrahimi et al. reported the imaginary (Im) *χ*^3^ value of GO in ethanol at 532 nm, which was 2.17 × 10^−14^ m^2^/V^2^ [37]. The values of (Im) *χ*^3^ change with GO dispersion in different solvents at 532 nm that varied from 1.0 × 10^−19^ to 5.1 × 10^−19^ m^2^/V^2^ [31]. In addition, Khanzadeh revealed that the *χ*^3^ of GO was 5.12 × 10^−16^ m^2^/V^2^. At the same wavelength, Kang et al. demonstrated that the *χ*^3^ value of GO is 8.97 × 10^−18^ m^2^/V^2^ [27]. It implies that the different magnitude of *χ*^3^ arises from the oxygen content in the sample, the types of solvent used, and the applied wavelength [38]. Our estimation of *χ*^3^ at 532 nm is one order higher than the one investigated using a continuous wavelength from 450 to 750 nm as per the literature review [27]. Furthermore, this study exhibits a positive nonlinear absorption coefficient, indicating that the excited state absorption cross-section surpasses that of the ground state. This characteristic makes it a promising candidate for utilization as an optical power limiter. It is worth noting that the highly intense laser beam employed in the nonlinear experiments may introduce a thermal effect into the results obtained from the z−scan [39]. Therefore, this article has provided a comprehensive analysis of both the polarity and magnitude of the nonlinear absorption and nonlinear refraction coefficients of graphene oxide (GO) in DI water. These measurements were conducted using a nanosecond laser emitting at a visible wavelength of 532 nm in a z−scan setup.

## 2. Materials and Methods

**Figure 1:**
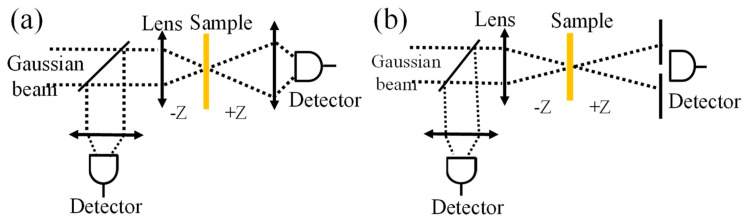
Schematic diagram of open (**a**) and closed (**b**) z−scan setups for characterizing the magnitude and polarity of nonlinear absorption and nonlinear refraction, respectively [41].

The GO nanoflakes in an aqueous solution were purchased from a graphene supermarket, Ronkonkoma, NY [40]. According to the vendor’s specifications, the GO product consists of more than 80% monolayers with nanoflakes ranging in size from 0.5 to 5 microns (https://www.graphene-supermarket.com/, accessed on 10 February 2023). The experiment utilized diluted GO, which was prepared using a standard 10 mm cuvette with a capacity of approximately 3.5 mL for dilution. The process involved mixing approximately 1.75 mL of the GO sample, which had a concentration of 6 g/L, with an equal volume of DI water. The third-order nonlinear optical properties of GO nanoflakes were characterized using the z−scan method in a 1 mm quartz cuvette. The excitation source for the z−scan was a pulsed laser at 532 nm, 10-Hz repetition rate, and ~6−ns temporal pulse width with the Gaussian beam [41]. The effective focal length of the focusing lens for the z−scan was ~125 mm. The radius of the beam waist (w_0_) at the focal point was ~15.1 µm. The Rayleigh length ($z_{0}=kw_{0}^{2}/2$) was ~2.02 mm, which was larger than the sample thickness (1 mm). The Gaussian beam at the focusing lens was ~2.8 mm at FWHM and was prepared using the two-irises method [41]. To ensure the integrity of our experimental setup, we meticulously eliminated all potential optical nonlinearities originating from electronic devices and other optical components by conducting experiments within the system’s linear range. Additionally, we performed z−scan experiments using only the base solvent to eliminate any undesired supplementary nonlinear effects from the sample. For validation purposes, we employed the nonlinear refraction of CS_2_ to verify the experimental setup through the closed z−scan technique [41]. The schematic diagrams of open and closed z−scan setups are depicted in Figure 1a and 1b, respectively [26], and have been adapted with permission from the previous publication [41].

## 3. Results and Discussion

**Figure 2:**
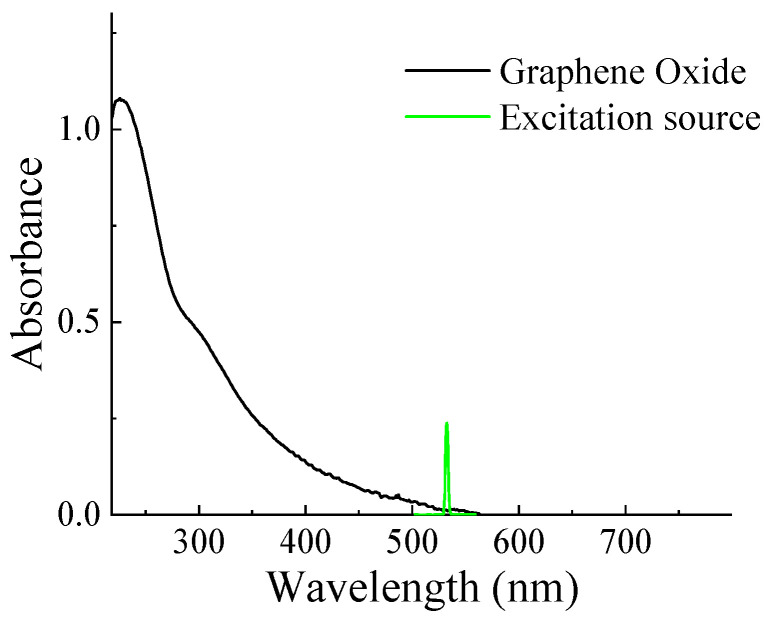
The absorption spectrum of GO nanoflakes in DI water. The green spectrum indicates the laser excitation used for nonlinear optical characterization.

In the linear absorption spectrum of GO nanoflakes in DI water, the absorption peaks are evident at around 230 nm and 300 nm, with their spectral tails extending into the visible region, as illustrated in Figure 2. The p orbitals of carbon can be combined in two ways: in-phase, resulting in bonding combinations, and out of phase, leading to anti-bonding combinations. This results in the formation of *π* and π* orbitals, with the *π* orbital having lower energy compared to the π* orbital. Consequently, this energy difference facilitates photon-induced transitions between the *π* and π* orbitals. The initial peak corresponds to the *π*–π* transition of the C=C bond, while the subsequent peak corresponds to the n–π* transition involving the carbonyl (C=O) bonds of GO [42].

**Figure 3:**
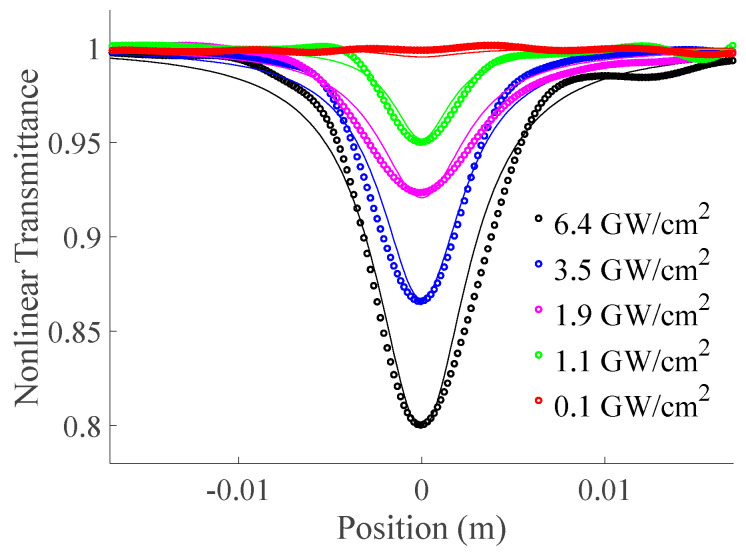
Normalized nonlinear transmittance as a function of the sample position (z) through the open z−scan technique, encompassing various applied peak intensities.

The nonlinear absorption behavior of GO nanoflakes in DI water was characterized through an open z−scan technique, revealing normalized nonlinear transmittance as a function of sample position (z) across various peak excitation intensities, namely ~6.4 GW/cm^2^, 3.5 GW/cm^2^, 1.9 GW/cm^2^, 1.1 GW/cm^2^, and 0.1 GW/cm^2^ at the focal plane (refer to Figure 3). These nonlinear transmittance traces distinctly exhibit a reverse saturable absorption (RSA) pattern, indicative of a positive nonlinear absorption coefficient [14]. This suggests that the absorption cross-section in the excited state surpasses that of the ground state. At lower wavelength regions, there is a notable increase in absorption, indicating the potential occurrence of two-photon excitation or two-photon absorption within the framework of optical nonlinearity. The normalized nonlinear transmittance via an open z−scan for a Gaussian beam is governed by [16].

(1) $$T(z,S=1)=\sum_{m=0}^{\infty }\frac{{\left(\frac{-q}{1+{\left(z/z_{o}\right)}^{2}}\right)}^{m}}{{\left(1+m\right)}^{3/2}}$$

where *z* is the sample position, $S=1-\exp\left(-2r_{a}^{2}/w_{0}^{2}\right)=1$ is the unit linear transmittance indicating no aperture in front of the detector in the open z−scan, *r_a_* is the radius of a finite aperture, *q*(*r*,*z*,*t*) = *βI_o_L_eff_* < 1 is the requirement for the open z−scan, which results in the negligible nonlinear phase distortion of $\Delta \Psi_{o}(t)=\beta I_{o}(t)L_{eff}/2$, $L_{eff}=\left(1-\exp\left(-\alpha_{o}L\right)\right)/\alpha_{o}L$ is the effective sample length, *L* is the sample thickness, *I_o_* is the applied peak intensity, *α_o_* is the linear absorption coefficient, and *β* is the nonlinear absorption coefficient. By fitting it to the model Equation (1), we estimate the nonlinear absorption coefficient of GO to be approximately ~2.62 × 10^−8^ m/W.

**Figure 4:**
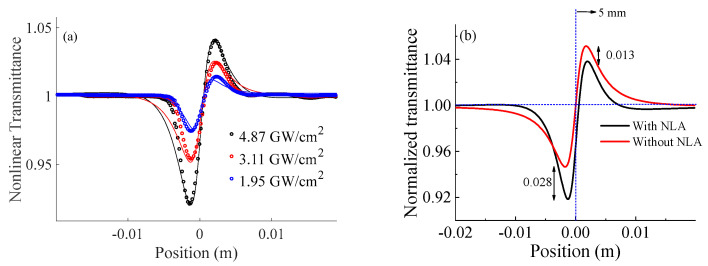
(**a**) Experimental normalized transmittance as a function of sample position (z) obtained through the closed z−scan technique, along with their corresponding fitting traces. (**b**) A comparative analysis of traces with and without the nonlinear absorption (NLA) coefficient at an applied intensity of 4.87 GW/cm^2^.

Furthermore, the normalized nonlinear refraction traces are characterized by closed z−scan techniques as shown in Figure 4a. It satisfied the far-field condition of an aperture (*d*~2.0 >> *z_o_*). The radius (*r_a_*) of a finite aperture is selected at ~0.75 mm to satisfy the linear transmittance of the finite aperture (*S*)~0.01 < 1 condition [41]. The normalized transmittance, depicted as a function of the sample position, showcases the presence of valley-peak traces, as exemplified in Figure 4. It suggests the self-focusing characteristics or positive nonlinear refraction observed in GO nanoflakes. The theoretical model of normalized nonlinear transmittance using a closed z−scan with a Gaussian beam is [26,43].

(2) $$T\left(S<<1\right)=1-\frac{4\Delta \varphi_{0}x+q\left(3+x^{2}\right)}{\left(1+x^{2}\right)\left(9+x^{2}\right)}-\frac{4\Delta \varphi_{0}^{2}\left(5-3x^{2}\right)-8\Delta \varphi_{0}qx\left(9+x^{2}\right)-q^{2}\left(40+17x^{2}+x^{4}\right)}{\left(1+x^{2}\right)\left(9+x^{2}\right)\left(25+x^{2}\right)}+\ldots$$

where *w_a_* is the radius of beam waist at the focal plane, $q\left(r,z,t\right)=\beta I_{o}L_{eff}<1$, $\Delta \varphi_{o}=k\gamma I_{o}L_{eff}<1$ is the phase distortion for the symmetric peak-valley nonlinear transmittance trace, *γ* is the nonlinear refraction coefficient, and $x=-\left(1/z_{o}\right)\left(z+\left(z_{o}^{2}+z^{2}\right)/d-z\right)\sim z/z_{o}$ for the far-field condition of an aperture (*d >> z_o_*), where *d* is the distance between the focal plane and the aperture. The nonlinear refraction coefficient (*γ*) is found to be ~3.9 × 10^−15^ m^2^/W from fitting with model Equation (2). Looking into the valley-peak transmittance, the valley is much deeper (~0.028 *T_v-p_*) than the valley-peak symmetry. This implies that absorption plays a considerable role in nonlinear refraction [44].

The NLR curve governed by Equation (2) results in transmittance variation between the normalized peak and valley *T_p_*_-*v*_ = 0.4|Δ*ϕ_o_*| and a peak–valley separation of *z_p_*_-*v*_ = 1.79 *z*_0_. Also, Δ*T_p-v_* depends on the magnitude of the nonlinear phase shift (Δ*ϕ_o_*) and the linear transmittance of the finite aperture (*S*). For a smaller phase shift (Δ*ϕ_o_* ≤ *π*), which resembles this experiment, the Δ*T_p-v_* follows the equation within ±2% variation [16].

(3) $$∆T_{p-v}\approx 0.406{\left(1-S\right)}^{0.25}|∆\phi_{o}|$$

For an applied intensity, the phase shift is constant. Therefore, this article is focused on investigating the effect of *S* on Δ*T_p-v_* using the estimated nonlinear refraction coefficient for three different applied peak intensities.

**Figure 5:**
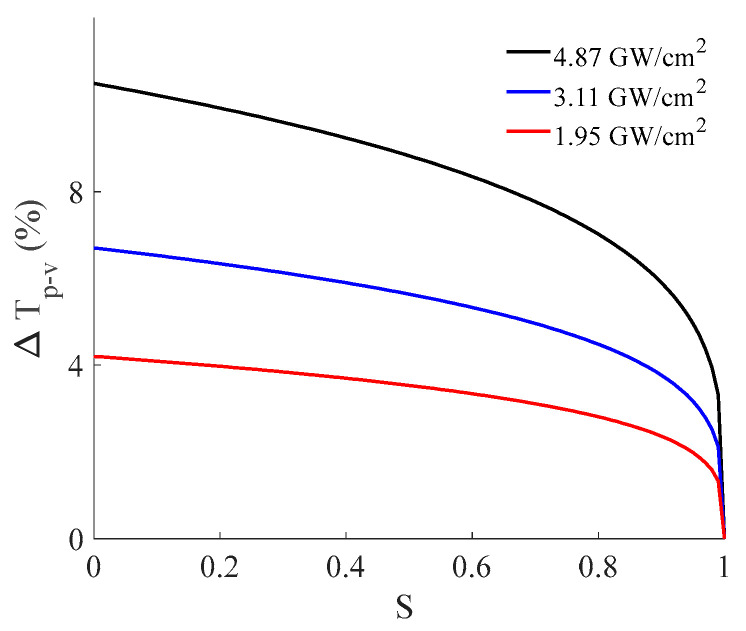
Difference between the normalized peak and valley transmittance as a function of linear transmittance of finite aperture (*S*) for different applied intensities.

**Figure 6:**
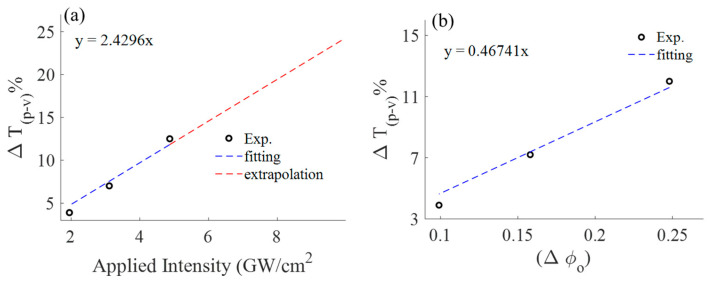
Percentage difference between the normalized peak and valley transmittance as a function of (**a**) applied intensity and (**b**) nonlinear phase shift (Δ*ϕ_o_*) for linear transmittance of finite aperture (0.01).

As depicted in Figure 5, it is evident that the magnitude of the peak-valley difference (Δ*T_p-v_*) decreases as the applied intensity decreases, which aligns with our expectations. Additionally, Δ*T_p-v_* diminishes in magnitude as the linear transmittance of the finite aperture increases, eventually reaching a point where Δ*T_p-v_* equals zero at *S* = 1. It is important to note that *S* = 1 represents the condition without an aperture, a setup typically used for investigating nonlinear absorption via the open z−scan technique. This suggests that selecting a smaller *S* value is the optimal condition for studying the phenomenon of nonlinear refraction. In our experiments, we conducted tests under three distinct applied intensities, each resulting in corresponding phase differences for a fixed *S* = 0.01. Figure 6 presents the magnitude of the peak-valley difference as a function of applied intensity (a), along with its fitting, and illustrates the nonlinear phase shift (b).

**Figure 7:**
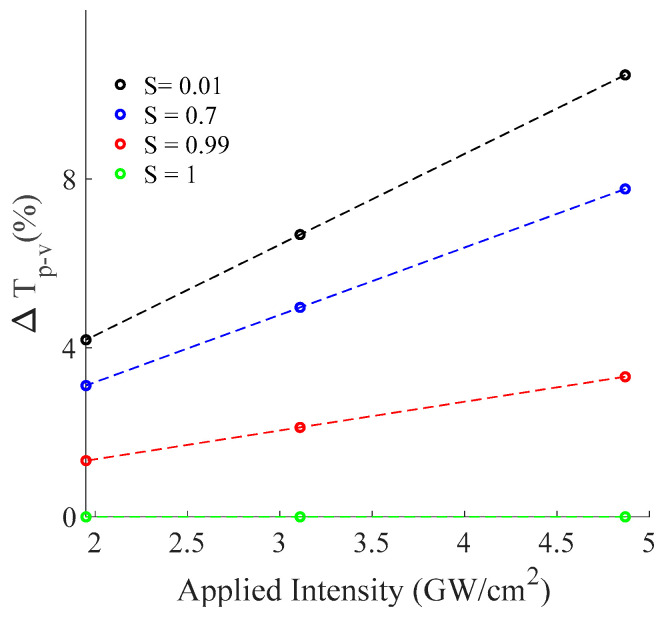
Percentage difference between the normalized peak and valley transmittance as a function of applied intensity for various linear transmittance of finite aperture (*S*).

Figure 6b displays the slope of Δ*T_p-v_* as a function of the phase shift at the focus point. The estimation yields a linear coefficient of ~0.46 which aligns with z−scan model [16]. In addition, for a higher *S*, the linear coefficients (slopes) become smaller, which is expected in the z−scan as shown in Figure 7 [16].

The absolute value of third-order nonlinear susceptibility is calculated using

(4) χ3=Reχ32+Imχ32

where Reχ3=4/3no2εocγ and Imχ3=1/3πno2εocλβ are real and imaginary components of third-order nonlinearity, *n_o_* is the linear refractive index [42], *ε_o_* is the dielectric constant of the vacuum, and *c* is the velocity of light. The third-order nonlinear susceptibility of the GO monolayer in DI water was estimated to be |*χ*^(3)^|~9.55 × 10^−17^ m^2^/V^2^, which is close to those in the literature review (~8.97 × 10^−18^ m^2^/V^2^ GO in DI water [27] and ~5.12 × 10^−16^ m^2^/V^2^ GO in ethanol [31]).

**Figure 8:**
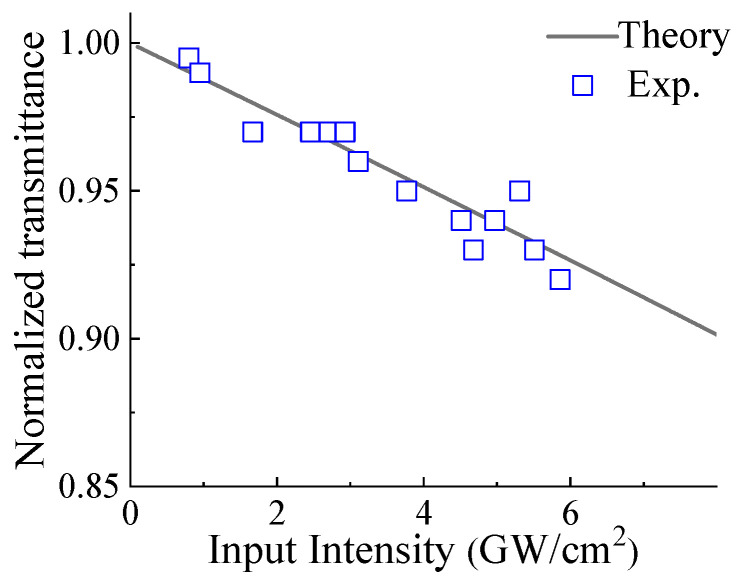
Normalized transmittance as a function of applied peak intensity at the valley position of valley-peak of closed z−scan traces.

The rise time of a thermal lens in an aqueous liquid is determined using the acoustic transit time, denoted as *τ* = *w*_o_/*v*_s_ [16] where *v*_s_ ≈ 1437 m/s [45], is the velocity of sound in the water at room temperature. Consequently, we calculate τ to be approximately 10.1 ns, nearly double the pulse width of around 6 ns. Furthermore, it is worth noting that the thermal diffusion time in water exceeds the pulse width [46]. It implies that there is non-uniform heating induced by the 6 nm pulsed laser, leading to a nonlinear index change at the focus axis. Consequently, it suggests that the third-order nonlinear optical response encompasses both electronic effects (Kerr effect) and thermal effects contributing to *γ*. The validity of the nonlinear refraction coefficient’s polarity is additionally confirmed through the measurement of transmittance as a function of applied intensity, employing the I−scan technique, as illustrated in Figure 8. The transmittance observed in the I−scan is contingent on multiple factors, including the applied intensity, nonlinear absorption, nonlinear refraction, sample position, Rayleigh range, effective sample length, and the distance between the sample and the aperture [16]. In the I−scan, the GO dispersion was positioned at the valley of the closed z−scan traces, which revealed a reduction in transmittance with increasing applied intensity. This phenomenon occurs because the transmittance beam experiences increased diffraction as the peak intensity rises. At the valley position, the intensity-dependent total absorption (*α*(*I*) = *α*_o_ + *βI*) and total refraction (*n*(*I*) = *n*_o_ + *γI*) collectively contribute to the laser power attenuation capacity, signifying the potential of GO nanoflakes as valuable materials for optical power limiting. Such materials can play a critical role in safeguarding the eyes and sensors from high-power radiation.

## 4. Conclusions

The third-order optical nonlinear properties of GO were studied through the z−scan technique using a single wavelength at a 532 nm laser source. The positive nonlinear (reverse saturable) absorption and nonlinear (valley-peak) refraction properties were observed through the open z−scan and closed z−scan techniques, respectively. Given the linear absorption spectra below the 500 nm region, the 532 nm excitation source likely facilitated nonlinear excitation through either two-photon excitation or a two-step excitation process, leading to reverse saturable absorption. This finding indicates that graphene oxide possesses a larger excited-state absorption cross-section than its ground state, making it a promising candidate for optical limiters. The estimated coefficients for nonlinear absorption and nonlinear refraction were approximately ~2.62 × 10^−8^ m/W and 3.9 × 10^−15^ m^2^/W, respectively. Moreover, we observed a reduction in laser transmittance through graphene oxide at higher intensities using an I−scan, suggesting its potential application in optical power-limiting devices in future technological advancements.

## Data Availability

The data presented in this study are available on request from the corresponding author.
